# ADAMTS6: Emerging roles in cardiovascular, musculoskeletal and cancer biology

**DOI:** 10.3389/fmolb.2022.1023511

**Published:** 2022-10-19

**Authors:** Timothy J. Mead

**Affiliations:** ^1^ Department of Pediatrics, Case Western Reserve University School of Medicine, Cleveland, OH, United States; ^2^ University Hospitals Rainbow Babies and Children’s Hospital, Cleveland, OH, United States

**Keywords:** ADAMTS6, matrix metalloprotease, extracellular matrix, cardiovascular, musculoskeletal, cancer

## Abstract

ADAMTS family members control mammalian development and disease, primarily through their function as proteases, by regulation of extracellular matrix composition. Until recently, ADAMTS6 was known as one of the orphan proteinases of the nineteen-member family with a relatively unknown expression pattern and function. Emerging focus on this enzyme has started to uncover these unknowns and revealed a vast importance and requirement of ADAMTS6 in cardiovascular and musculoskeletal development. In addition, ADAMTS6 has been linked to numerous disease settings including several types of cancer. This review summarizes the necessity of ADAMTS6 during development, its role in disease and requirement for essential prospective studies to fully realize its biological implications and potential for therapeutic intervention.

## Introduction

A disintegrin-like and metalloprotease with thrombospondin type 1 motif (ADAMTS) genes encode secreted proteases that have demonstrated critical roles during adult tissue homeostasis and disease acting within the extracellular matrix (ECM) (Mead and Apte, 2018). Their roles during development are well addressed with many ADAMTS gene knockouts resulting in embryonic lethality or causative in disease (Dubail and Apte, 2015). The functional subdivisions or clades of the 19 family members is based upon common affinities towards preferred substrates and evolutionary homology (Brunet et al., 2015). They share similar domain organization with a common structure, yet distinct modules. They all have a propeptide region, an N-terminal protease domain, variable number of thrombospondin type 1 repeats (TSRs) with differing C-terminal domains. The biological functions of ADAMTS proteases are largely determined by their substrates and the subsequent actions or necessity of the substrates in biological processes. While many ADAMTS family members have been extensively studied *in vitro* and *in vivo* with identified substrates and mapped cleavage sites, such as in the case of multiple ADAMTS proteases cleaving the proteoglycans aggrecan and versican, other family members are not that well defined in terms of expression profile, necessity during tissue development and homeostasis or substrate identification (Verma and Dalal, 2011; Nandadasa et al., 2014; Santamaria, 2020; Martin et al., 2021).

ADAMTS6 maps to human chromosome 6 and mouse chromosome 13 and was first identified to be expressed in human placental tissue and the domain structures were subsequently identified (Hurskainen et al., 1999). Like other ADAMTS protease family members, ADAMTS6 is secreted as a zymogen and subsequently processed, and thus activated, by furin (Turpeinen et al., 2011; Cain et al., 2016). Protein *O*-fucosyltransferase-2 (POFUT2) is an enzyme that adds *O*-linked fucose to the TSRs, which are numerous in ADAMTS6, in the endoplasmic reticulum to promote trafficking of substrates that include ADAMTS proteases (Neupane et al., 2022). TSR1-3 were modified with the O-fucose disaccharide while TSR1 was additionally modified with C-mannose (Neupane et al., 2022). Notably, inactivation of *POFUT2* resulted in decreased ADAMTS6 secretion. Knockout and conditional knockout of *Pofut2* resulted in shortening of limbs and accumulation of fibrillin-2 microfibrils, which mirrored *Adamts6*-deficient mice (this will be discussed below) (Mead et al., 2022; Neupane et al., 2022). It was further suggested that ADAMTS6 does not undergo autocatalysis, as evident by lack of additional bands in western blot analysis and comparable band intensity to catalytically inactivated ADAMTS6 (Mead et al., 2022).

*Adamts6* is expressed in numerous vertebrates in a variety of tissues. In zebrafish, it is enhanced 200-500-fold after 8 h post fertilization and expressed in all organs surveyed including high expression in the liver, kidney and skeletal muscle as assessed by quantitative real-time PCR (qRT-PCR) (Brunet et al., 2015). Expression of *Adamts6* mRNA was shown by RNAscope *in situ* hybridization in murine tissues including cardiac outflow tract, heart valves, myocardium, cartilage, tendon, and skeletal muscle amongst other tissues (Prins et al., 2018; Mead et al., 2022). In human tissue, *ADAMTS6* is expressed in retinal pigment epithelial cells (RPE) as assayed by RT-PCR and northern blot analysis as well as adult chondrocytes (Bevitt et al., 2003; Kevorkian et al., 2004; Davidson et al., 2006).

It is also expressed in a variety of tumors from different origins and associated with other pathological situations as described in detail below. However, the role of ADAMTS6 in these and other yet undescribed tissues and disease settings are not fully uncovered. ADAMTS6 has long been considered an “orphan protein” with very little data on its expression profile, function, and substrate targets. Very recent studies have started to uncover these unknowns and provide a strong case for the necessity of ADAMTS6 in ECM maintenance during development and its implications during disease processes. The main processes that ADAMTS6 has been identified to participate in will be discussed in the following sections, spanning embryology through cancer and other disease settings.

## ADAMTS6 in cardiovascular development and disease

**FIGURE 1:**
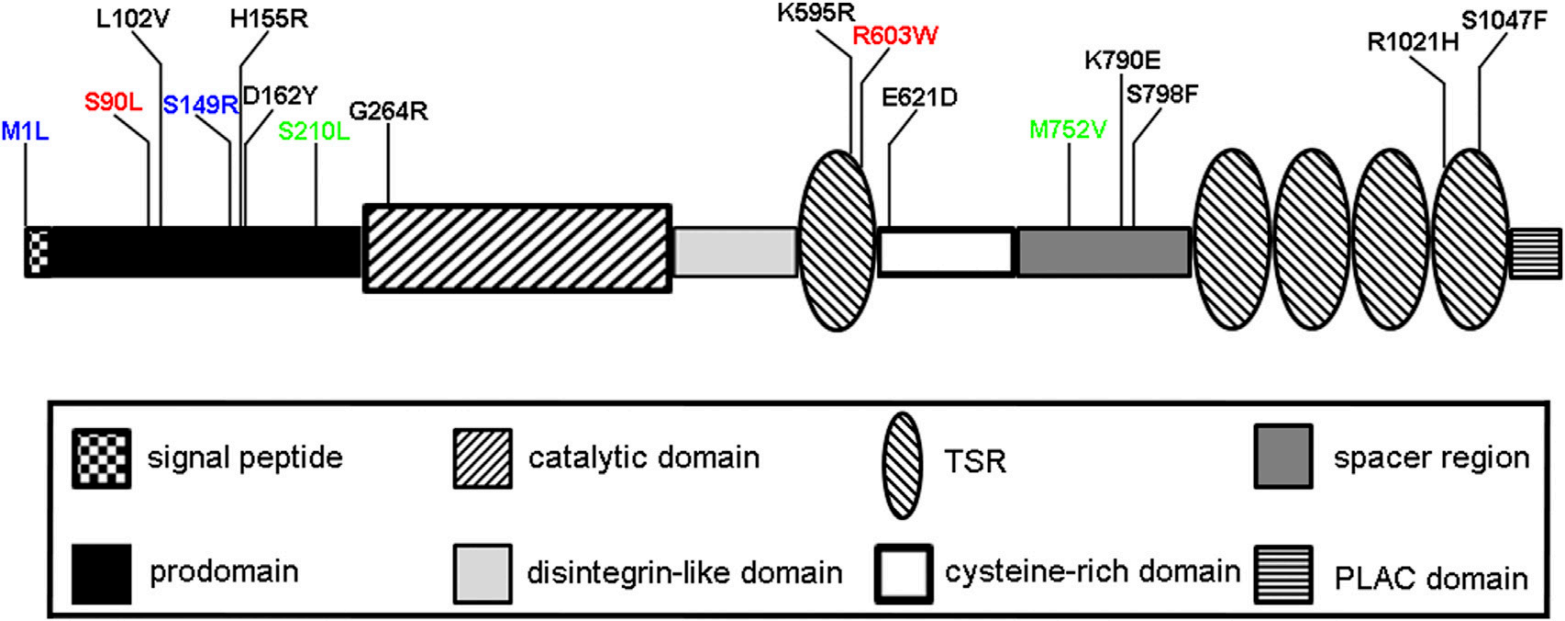
Schematic of ADAMTS6 domain structure and allelic mutations. Mutations shown in blue (M1L, S149R) are recessive mutations identified in mice that cause prenatal/neonatal lethality (Li et al., 2015). Two rare non-synonymous variants rs61736454 (p.Ser90Leu) and rs114007286 (p.Arg603Trp) are shown in red, predicted damaging variants in cardiac QRS interval are shown in black and benign-predicted variants (S210L, M752V) are shown in green (Prins et al., 2018).

*Adamts6* is expressed throughout the developing heart with strong staining noted in the outflow tract, valves and myocardium (Prins et al., 2018). As part of the “bench to bassinet program”, an ethylnitrosourea (ENU) mutagenesis forward screen uncovered two point mutations in *Adamts6* that result in late embryonic death likely due to defective cardiovascular development (Li et al., 2015). One mutation effected the start codon located at position 1 (p.Met1Ile) while the other was a Ser-Arg mutation at position 149 (p.Ser149Arg) in the prodomain, both resulting in lack of ADAMTS6 protein (Figure 1). A subsequent study showed that the p.Ser149Arg mutation, with a conserved serine residue across numerous species, resulted in no secretion in the medium in a cell culture assay effectively resulting in a knockout (Prins et al., 2018). These *Adamts6* ^S149R/S149R^ mice present with a myriad of congenital heart defects including double outlet right ventricle, persistent truncus arteriosus, defective atrial septation and ventricular hypertrophy (Prins et al., 2018). However, the cardiovascular targets of ADAMTS6 and the underlying mechanisms of its necessity during outflow tract development remain unknown and warrants further investigation.

Cardiac conduction is controlled by electrical impulses in a network of specialized cardiomyocytes that regulate ventricular contraction. If this is not properly regulated, cardiac conduction disorders, such as an arrhythmia, may occur. The duration of ventricular depolarization is noted by the QRS interval on an electrocardiogram. Prolonged QRS intervals are an independent predictor of mortality and commonly associated with left side heart failure (Desai et al., 2006; Badheka et al., 2013; Mozos and Caraba, 2015). A genome-wide association study uncovered rare, non-synonymous *ADAMTS6* variants that are associated with altered cardiac ventricular conduction, namely a prolonged QRS interval (Figure 1) (Prins et al., 2018). These predicted pathogenic mutations had reduced secretion from the cells into the medium as compared to predicted benign variants, resulting is diminished available ADAMTS6 protein and thus proteolytic activity. To this end, *Adamts6* ^S149R/S149R^ mice, which is considered an *Adamts6*-deficient mouse, had reduced connexin 43 staining in embryonic myocardium. *Adamts6* ^S149R/+^ juvenile and adult myocardium also had reduced connexin 43 staining as compared to control, indicative of reduced gap junctions, and is one likely explanation for defective cardiac contraction (Prins et al., 2018). As is with the outflow tract, the role of ADAMTS6 in myocardium, and in particular the cardiac conduction system, is not fully understood and might be aided by a conditional loss of function approach in mice.

## ADAMTS6 in musculoskeletal tissue development and disease

The role of ADAMTS6 in musculoskeletal tissue is starting to come into focus. *ADAMTS6* is highly expressed in both normal and osteoarthritic cartilage and synovium as identified from qRT-PCR analysis (Kevorkian et al., 2004; Davidson et al., 2006). Its expression is upregulated in primary annulus cells grown from degenerated human lumbar discs as shown by microarray analysis (Gruber et al., 2010). *ADAMTS6* expression was downregulated, as shown by RNA-seq and validated by qRT-PCR, in adipose-derived stem cells undergoing osteogenesis and a genome wide association study (GWAS) identified *ADAMTS6* as a promising, but not yet genome-wide significant, susceptible loci for osteosarcoma (Savage et al., 2013; Shaik et al., 2019).

A recent study illustrated the role of ADAMTS6 in musculoskeletal tissue utilizing a mouse model. RNAscope *in situ* hybridization showed *Adamts6* was detected not only in resting and proliferative chondrocytes, but also in the perichondrium, which lines the developing cartilage anlagen (Mead et al., 2022). Further expression was demonstrated throughout the embryonic musculoskeletal system including in the skeletal muscle, ligaments and tendons with strong expression noted in the joint regions. *Adamts6*-deficient mice die at birth due to defective cardiovascular development, however, they also present with appendicular skeletal abnormalities including club foot-like phenotype as well as a myriad of axial skeleton manifestations including shorter ribs, defective sternum ossification as well as facial deformities including micrognathia and cleft palate (Mead et al., 2022). *Adamts6* ^−/−^ embryos are runted and the forelimb and hindlimb bones are all shortened and widened as compared to control. The distal hindlimb tibia and fibula bones are additionally bent, with noted severe reduction of ossification in the fibula, which manifests in a club limb-like phenotype. There is an apparent delay of endochondral ossification and mineralization from embryonic day (E) 14.5 to E18.5 in the hindlimb bones with the fibula being the most drastic example. *Adamts6* ^−/−^ growth plates are disorganized and have a reduction of key chondrogenic markers, including aggrecan, cartilage link protein and Sox9 with no change in chondrocyte proliferation or cell death (Mead et al., 2022). Staining of Col X, a marker of hypertrophic chondrocytes, is enlarged and points to an expanded hypertrophic chondrocyte zone, possibly at the expense of other chondrocyte zones or bone formation.

Due to the malformation in the distal hindlimb, the *Adamts6* ^−/−^ knee joint was a point of further investigation. Disorganized and increased collagen staining is apparent in *Adamts6* ^−/−^ perichondrium and adjacent mesenchyme with increased staining of MAGP1 and fibrillin-2 (Mead et al., 2022). Overexpression of ADAMTS6 resulted in no apparent fibrillin-1, fibrillin-2 or fibronectin microfibrils in an *in vitro* model. Conversely, in the absence of *Adamts6* or in the presence of catalytically inactive ADAMTS6 (loss of protease activity), there were abundant microfibrils present, which points to a direct role of ADAMTS6 in microfibril formation or turnover. Therefore, these proteins were further explored as possible substrates. Direct binding of ADAMTS6 to fibrillin-1, fibrillin-2 and fibronectin was noted in Biacore assays and proteomic assays revealed that they were all indeed substrates through identification of specific cleavage sites (Mead et al., 2022). Reduced BMP, but not TGFβ signaling, was shown in *Adamts6* ^−/−^ hindlimb tissue as compared to control, pointing to a potential regulation of this major signaling pathway.

Due to an increase of fibrillin-2 staining in *Adamts6* ^−/−^ hindlimbs, absence of fibrillin-2 microfibrils in the presence of active ADAMTS6, direct binding of ADAMTS6 to fibrillin-2 and a specific cleavage site identified in fibrillin-2 due to ADAMTS6 activity, the relationship of ADAMTS6 and fibrillin-2 was further explored *in vivo*. *Fbn2* haploinsufficiency on the *Adamts6* ^−/−^ background (*Adamts6* ^−/−^;*Fbn2* ^+/−^) resulted in genetic reversal of limb abnormalities exhibited in *Adamts6* ^−/−^ mice with restoration of chondrocyte growth plate markers (Mead et al., 2022). Reversal of axial skeleton abnormalities were also evident. Notably, reduction of *Fbn1* did not affect *Adamts6* ^−/−^ skeletal defects pointing to a direct role of ADAMTS6 cleavage of fibrillin-2 in the musculoskeletal tissues as necessary for proper skeletal formation.

Beyond its recently discovered roles during musculoskeletal development and associated malformations in its absence, it is important to study the role of ADAMTS6 in postnatal musculoskeletal tissues. This can be achieved in a murine conditional loss of function approach in numerous musculoskeletal tissue-specific lines. ADAMTS6 has already been implicated in osteoarthritis and it could prove fruitful to study this in an osteoarthritis mouse model, for example.

## ADAMTS6 relationship to ADAMTS10

ADAMTS6 is a sister protease of ADAMTS10, based on the ADAMTS phylogenetic tree, with identical gene domain structures, high amino similarity of both the full length and catalytic domain (60% identical amino acid residues) (Apte, 2004; Somerville et al., 2004; Karoulias et al., 2020; Rose et al., 2021). Their relationship was explored in *in vitro* studies, utilizing human retinal pigmented epithelial cells. Knockdown of *ADAMTS10* resulted in an increase of *ADAMTS6* expression and overexpression of *ADAMTS10* resulted in decreased *ADAMTS6* expression, strongly suggesting that *ADAMTS10* negatively regulates *ADAMTS6* and that *ADAMTS6* can compensate for loss of *ADAMTS10* (Cain et al., 2016). Overexpression of *ADAMTS6* resulted in increased *ADAMTS10* while knockdown of *ADAMTS6* resulted in decreased *ADAMTS10*, suggestive of an *ADAMTS6* positive regulation of *ADAMTS10*. Their relationship was further explored *in vivo* using *Adamts6* ^−/−^ and *Adamts10* ^−/−^ embryonic limb, heart, and lung samples (Mead et al., 2022). In this study, transcriptional adaptation was apparent in that *Adamts6* was upregulated in *Adamts10* ^−/−^ samples while *Adamts10* was upregulated in *Adamts6* ^−/−^ tissues. The difference between the two studies in term of mutual regulation could be an *in vitro* versus an *in vivo* difference or cell type specific differences in how ADAMTS6 and ADAMTS10 interact. The finding of cooperation was demonstrated in double-knockout mice (*Adamts6* ^−/−^;*Adamts10* ^−/−^) which had additional musculoskeletal phenotypes compared to *Adamts6* ^−/−^ alone consisting of forelimb buckling in a similar manner to *Adamts6* ^−/−^ hindlimbs and subcutaneous hemorrhage, neither of which appear in *Adamts10* ^−/−^ mice (Mead et al., 2022). It would be of interest to investigate *Adamts6* ^−/−^;*Adamts10* ^−/−^ embryonic hearts to see if the *Adamts6* ^−/−^ congenital malformations worsen or new phenotypes arise as a result of concurrent loss of *Adamts10*, and therefore loss of compensation, in *Adamts6* ^−/−^ hearts.

## Emerging roles of ADAMTS6 in cancer

ADAMTS6, like many other ADAMTS family members, has a pro- or anti-tumor role in various types of cancer, spanning several organ systems. In some instances, *ADAMTS6* was shown to be either upregulated or downregulated in the same cancer type based on microarrays, clinical samples, and *in vitro* assays. White the overall majority of studies show that increased expression of *ADAMTS6* is pro-cancer and correlates with a negative outcome, there are a few exceptions noted.

A survey of ADAMTS family members showed that *ADAMTS6* expression is upregulated in breast carcinoma tissue samples with highest expression in myoepithelial cells (Porter et al., 2004). In agreement, a further study showed that elevated levels of *ADAMTS6* shown by RNA-seq and validated by qRT-PCR, *via NFATC2* upregulation, was demonstrated in a metastasized breast cancer cell line (Xu et al., 2021). Knockdown of *ADAMTS6* in this cancer cell line resulted in delayed wound healing and decreased cell migration. Furthermore, elevated levels of *ADAMTS6* mRNA associated with poor prognosis in breast cancer patients in overall survival (Xu et al., 2021). In a contrasting study, increased level of *ADAMTS6* was associated with breast cancer tumor suppression in numerous breast cancer cells lines (Xie et al., 2016). In the cancer cell lines surveyed, ADAMTS6 protein and mRNA levels were downregulated. Artificially enhanced *ADAMTS6* correlated with suppression of breast cancer cell migration, invasion and tumorigenesis and associated with disease-free survival. This favorable finding was in part mediated through regulation of the ERK signaling pathway through decreased phosphorylation and ADAMTS6 was shown to bind a microRNA, which reduced its expression (Xie et al., 2016).

ADAMTS6 has been linked to non-small cell lung cancer (NSCLC) through regulation of anterior gradient 2 (AGR2), an endoplasmic reticulum protein family member (Luu et al., 2020). In this cancer, tumor cell-acquired drug resistance is problematic, especially in patients who initially respond favorably to epidermal growth factor receptor tyrosine kinase inhibitors (EGFR-TKI) and subsequently acquire resistance to treatment (Pao et al., 2005). *AGR2* is upregulated in drug-resistant NSCLC tumor cells and corelated with cancer cell proliferation, migration, and invasion. Knockdown of *AGR2* resulted in increased expression of *ADAMTS6* as well as decreased *VEGFA*, a marker of vascular endothelium and surrogate marker of angiogenesis (Luu et al., 2020). Importantly, knockdown of *ADAMTS6* resulted in upregulation of AGR2 mRNA and protein and provides the evidence for a negative feedback loop in these NSCLC cells. The relationship of ADAMTS6 and NSCLC was further delineated by another group, focusing on epigenetic regulators of histone methylation. Chromatin immunoprecipitation and qRT-PCR analysis revealed that *ADAMTS6* expression is over 60-fold upregulated during epithelial to mesenchymal transition (EMT) and associated with loss of H3K27me3 on its promoter (Lachat et al., 2020). Increased ADAMTS6 staining was more evident in EMT+ tumors than in EMT- tumors while knockdown of *ADAMTS6* decreased cell invasion (Lachat et al., 2020). Therefore, the authors concluded that increased *ADAMTS6* expression mediated by H3K27 demethylation favored invasion during EMT.

Bulk RNA-Seq identified *ADAMTS6* as one of the most differentially expressed genes in a survey of data sets of human colorectal cancer as compared to normal tissue (Xiao et al., 2015). Further qRT-PCR validation revealed that *ADAMTS6* is significantly downregulated in colon cancer while significantly upregulated in rectal cancer (Xiao et al., 2015). This dichotomy points to differing regulatory mechanisms and signaling pathways at work in colon and rectal cancer (Li et al., 2012). A subsequent study, utilizing RNA-Seq data and qRT-PCR analysis, identified *ADAMTS6* as highly expressed in colon cancer tissues and cell lines and was associated with cancer progression and predicted worse prognosis in colon cancer patients (Wang et al., 2020). Knockdown of *ADAMTS6* in a colon cancer cell line resulted in repression of colon cancer cell growth, invasion, and migration while overexpression led to the opposite. This was in part mediated by the regulation of E-cadherin, N-cadherin, Vimentin, Snail p-AKT and p-p65 and point to the regulation of EMT and AKT/NFκB signaling pathways, respectively (Wang et al., 2020). Therefore, ADAMTS6 might act as a catalyst for EMT in colon cancer progression thereby enhancing colon cancer cell growth, proliferation, migration, and invasion. Further studies are warranted to fully understand the role of ADAMTS6 in colon cancer cell biology.

A study utilizing The Cancer Genome Atlas (TCGA) and RNA-seq data revealed that high *ADAMTS6* expression in gastric cancer patients associated with poor clinical outcome and reliably predicted overall survival (Zhu et al., 2021). Consistent with this finding, immunohistochemistry and qRT-PCR analysis showed increased ADAMTS6 protein staining and mRNA expression, respectively, in gastric cancer samples. Furthermore, *ADAMTS6* expression is correlated with tumor stage and histological grade and shown to promote the development and occurrence of stomach cancer (Zhu et al., 2021). Gene set enrichment analysis showed that high *ADAMTS6* mRNA levels led to elevated VEGF and TNF pathway expression amongst other cancer-related pathways. Therefore, elevated levels of *ADAMTS6* in gastric cancer is a tumor promoter and mediates occurrence, tumor cell proliferation, invasion and metastasis that leads to increased mortality.

Several other cancers have been linked to ADAMTS6. *ADAMTS6* was significantly downregulated in placental site trophoblastic tumor (PSTT), a rare type of gestational trophoblastic neoplasms through interrogation of microarray data and confirmed by qRT-PCR analysis (Gan et al., 2022). This finding was further validated *via* immunohistochemistry with diffuse ADAMTS6 positive staining in normal villi while reduced staining was apparent in PSTT cells (Gan et al., 2022). Increased staining of ADAMTS6 is an indicator of poor prognosis in patients with esophageal squamous cell carcinoma (Liu et al., 2018). Shown to be abundantly apparent in the cytoplasm and nucleus, ADAMTS6 showed strong staining in the cancer cell invasive front. While it was not shown to be correlated with the patient’s age, sex or tumor size, ADAMTS6 staining was corelated with clinical stage, metastasis, recurrence and poor overall survival (Liu et al., 2018). A further study utilizing qRT-PCR analysis showed increased expression of *ADAMTS6* in aggressive and aggressive-invasive prolactin pituitary tumors, as compared to non-invasive tumors, and further showed that this increase in expression corelated with tumor recurrence and progression (Wierinckx et al., 2007; Raverot et al., 2010).

With the dual role of ADAMTS6 as either cancer tumor suppressor and pro-tumoral agent, analysis and correlation studies are of key importance to decipher the precise role of ADAMTS6 in cancer development. The actions of ADAMTS6 may depend upon several factors including the type of cells present as well as the interactions with other ECM components. Further studies that add to current *in vitro* data, such as inhibiting or enhancing ADAMTS6 in tumor mouse models is warranted to study these cancers in depth to reveal therapeutic potential.

## ADAMTS6 in disease

Along with ARID1B, disruption of ADAMTS6 is associated with developmental delay, speech impairment, dysmorphic features and intellectual learning disability (Malli et al., 2014). The genomic translocation breakpoint identified that a sequence from intron 11 of *ADAMTS6* fused to *ARID1B* intron 2. Whether this genomic translocation is a result of either ADAMTS6 or ARID1B or both being disrupted is unknown. While a few patients with developmental delay have been identified with large deletions in the ADAMTS6 locus, ARID1B is a known regulator of neural development and has previously been associated with intellectual disability (Firth et al., 2009; Halgren et al., 2012; Hoyer et al., 2012; Santen et al., 2012; Tsurusaki et al., 2012). This warrants further investigation.

Recently, ADAMTS6 has been linked to many microRNAs (miR) in several disease settings. *ADAMTS6* was down regulated by miR-210-3p in a screen of preeclampsia biomarkers utilizing the Gene Expression Omnibus database (Jiang et al., 2020). It was further shown that Inc-CTD2383M3.1 acted as a competing endogenous RNA by competitively binding to miR-210-3p, thus alleviating suppression of *ADAMTS6* (Jiang et al., 2020). A further study, utilizing bioinformatics, showed that ADAMTS6 is a potential hub for miRNAs of oral fluids related to orthodontics (Kapoor et al., 2021). Bioinformatics, RNAseq and qRT-PCR analysis, along with *in vitro* studies, showed that circular (circ)*ADAMTS6* RNA, a type of noncoding RNA, was downregulated after treatment of IL-1β (Fu et al., 2021). *circADAMTS6*, originated from the ADAMTS6 gene and located in cytoplasm, is a 669 nucleotide circRNA that was formed from joining the 5′ splice site of exon 21 to the 3’ splice site of exon 24 (Fu et al., 2021). Overexpression of *circADAMTS6* inhibited human chondrocyte apoptosis while overexpression of miR-431-5p had an opposing effect. Suggested binding of *circADAMTS6* to miR-431-5p was shown through RNA-FISH and luciferase assays showing both located in direct contact in the cytoplasm, although definitive studies determining their relationship need to be explored further. Another connection of *circADAMTS6* to a miRNA was explored in a follow up osteoarthritis study. qRT-PCR analysis showed that *circADAMTS6* expression is reduced in osteoarthritic cartilage as compared to normal control cartilage (Shen et al., 2022). Knockdown of *circADAMTS6* reduced chondrocyte proliferation and increased apoptosis and elevated inflammatory response in control human cartilage, which mirrored IL-1β stimulation in normal cartilage. Overexpression of *circADAMTS6* with concurrent inhibition of miR-324-5p enhanced human chondrocyte proliferation while suppressing chondrocyte apoptosis, inflammatory response and the PI3K/AKT/mTOR pathway (Shen et al., 2022). Taken together, *circADAMTS6* negates IL-1β-induced chondrocyte apoptosis and warrants further studies in the treatment of osteoarthritis. Up regulation of another circ*ADAMTS6* was associated with poor overall survival in patients with esophageal squamous cell carcinoma (ESCC) (Bu et al., 2022). Conversely, knockdown of circ*ADAMTS6* inhibited ESCC cell proliferation, migration and invasion in xenograft tumors. Thus, as with the role of ADAMTS6 in cancer, the role of circ*ADAMTS6* in disease is tissue specific.

Additional disease settings involving ADAMTS6 have been reported, but have limited data and require follow up studies. *ADAMTS6* was upregulated, as assessed by microarray and confirmed by qRT-PCR analysis, in kidney epithelial cells in an *in vitro* model of Fabry disease, an X-linked lysosomal disorder (Shin et al., 2015). *ADAMTS6* is highly expressed in osteoarthritic cartilage and synovium as identified from qRT-PCR analysis (Kevorkian et al., 2004; Davidson et al., 2006). Two GWAS identified *ADAMTS6* as a susceptible locus for inguinal hernia (Jorgenson et al., 2015; Fadista et al., 2022). *Adamts6* expression, as determined by RNA-seq and qRT-PCR analysis, was noted in mouse connective tissue equivalent to human transversalis fascia, which when weaken or defective can lead to an inguinal hernia (Jorgenson et al., 2015). And finally, GWAS located single nucleotide polymorphisms (SNPs) in the region of *ADAMTS6* associated with changes in central corneal thickness in the eye and may lead to disease if not properly maintained (Lu et al., 2013).

## ADAMTS6 substrates

Like other family members, ADAMTS6 participates in ECM remodeling and thus targets will likely be found in this area. Evidence that fibrillin-1, fibrillin-2 and fibronectin were potential substrates was shown through increased immunofluorescence staining in *Adamts6* ^−/−^ hindlimbs, as compared to control, with no change in RNA levels (Mead et al., 2022). They were therefore subjected to BIAcore analysis and shown to all bind to ADAMTS6 and followed up by targeted proteomic searches in which the substrate of interest was overexpressed in the presence or absence of active ADAMTS6, which identified specific cleavage sites in each. Cleavage of fibrillin-2 was verified through western blot analysis that showed cleavage products when the C-terminal half of fibrillin-2 was co-cultured with ADAMTS6 overexpressing cells, but not in the presence of inactivate ADAMTS6 (Mead et al., 2022). In a series of experiments utilizing retinal epithelial cells, Biacore analysis revealed that ADAMTS6 binds to heparin, syndecan-4, fibrillin-1 and LTBP-1 (Cain et al., 2016). ADAMTS6 was shown to cleave LTBP-1 in a purified protein to protein assay to show direct interaction, but the precise cleavage site is unknown (Cain et al., 2016). The cleavage of LTBP-1 by ADAMTS6 suggested a potential role in regulation of TGFβ signaling. While TGFβ signaling, as measured by pSMAD1/5/8 staining, was unchanged in *Adamts6*-deficient embryonic hindlimbs, another tissue and setting could uncover a yet unknown role and function (Mead et al., 2022). Western blot analysis also showed cleavage of syndecan-4 by ADAMTS6, but no cleavage site was determined (Cain et al., 2016).

While these few ADAMTS6 substrates have been suggested and fibrillin-2 verified through cleavage site identification and genetic interaction, additional focus on substrate verification and identification is warranted. *ADAMTS6* is expressed in numerous cell types and tissues during development and disease and its roles in these settings will not be fully realized until additional tissue-specific substrates are identified. Thus, an unbiased proteomic search in select ECM-enriched populations is necessary to uncover additional substrates. Once these cleavage sites are verified and shown to be critical, utilizing *in vitro* and *in vivo* means, only then can the role of ADAMTS6 in these settings be realized which is necessary to reveal new avenues of research towards therapeutic intervention.

## Conclusion and perspectives in ADAMTS6 functional biology

This review summarizes the known ADAMTS6 connections to development and diseases known currently. This will undoubtedly expand in the near future and beyond as this once “orphan protein” starts to be recognized and more studies are undertaken, such as whole genome sequencing of rare disorders in humans and preclinical models as well as proteomic screenings. ADAMTS6 participates in a myriad of processes including cardiac and musculoskeletal development as well as cancer and disease and interacts with many ECM molecules and signaling pathways as a result. Greater understanding of the ADAMTS6 regulatory network is vital to predict undesired outcomes when attempting to modify consequences of disease.

*Adamts6*-deficent mice have made a considerable impact on the ECM field and have led to the discovery of its necessity in cardiovascular and musculoskeletal biology (Li et al., 2015; Prins et al., 2018; Mead et al., 2022). However, the mice die at birth and thus preclude investigation into any postnatal studies or reveal further complications from absence of *Adamts6* in disease settings. *Adamts6* is expressed in differing cells lineages and it is difficult at present to discern cell type specific functions. Therefore, a conditional genetic approach is warranted in future *in vivo* studies. The full picture of the role of ADAMTS6 can only begin to be realized once the substrates are identified and verified. This will allow for delineation of molecular pathway and mechanistic insight into its role in the ECM during tissue development, homeostasis, and disease settings. While a few substrates have been suggested and some verified, an unbiased, proteomics approach is warranted to discern the full landscape. Only then will it be readily suitable to generate targeted approaches for therapeutic applications.

Fibrillin microfibrils are a necessary component of the ECM that provides mechanical stability to tissues and regulates several signaling pathways such as TGFβ and BMP (Ramirez and Rifkin, 2009). That ADAMTS6 regulates fibrillin microfibrils in development warrants further investigation in disease settings. ADAMTS6 was shown to bind to and cleave both fibrillin-1 and fibrillin-2, yet only fibrillin-2 increased abundance was apparent in *Adamts6* ^−/−^ musculoskeletal tissues during development (Mead et al., 2022). Whether that holds true in other tissues during development or in adult tissue homeostasis or disease remains to be explored. Fibrillin microfibrils can form homodimers or heterodimers and it is possible that the dominance of *fbn1* postnatal expression as compared to *fbn2*, which is virtually absent starting in early postnatal tissues, is a result of abundance fibrillin-1 transcription masking a fibrillin-2 microfibril core (Zhang et al., 1995; Lin et al., 2002; Charbonneau et al., 2003; Marson et al., 2005). Therefore, it is possible that ADAMTS6 targets fibrillin-1 predominantly in postnatal tissues but may also target fibrillin-2 if unmasked. This will be necessary to explore and understand if targeting one versus the other is preferred in a disease setting.

A further function of ADAMTS6 was uncovered in focal adhesion biology. Through a series of experiments utilizing a human retinal epithelial cell line, it was shown that ADAMTS6 negatively regulates focal adhesions (Cain et al., 2016). Addition of the C-terminal half of ADAMTS6 stimulated adhesions and was bound to heparin and syndecan-4, both focal adhesion components (Woods et al., 2000). Overexpression of full length, active ADAMTS6 resulted in lack of focal adhesions while ADAMTS6 knockdown or mutations in the ADAMTS6 catalytic domain or in the furin cleavage site (both mutations rendering ADAMTS6 inactive) resulted in an over-abundance of focal adhesions (Cain et al., 2016). Additionally, heparin sulfate, which is necessary for induction of focal adhesions (Gopal et al., 2010), was depleted in the presence of ADAMTS6 and abundant in ADAMTS6 knockdown cells as determined by a prominent cell surface glycocalyx (Cain et al., 2016). The lack of focal adhesions in the presence of ADAMTS6 influenced microfibril deposition. Overexpression of ADAMTS6 resulted in lack of microfibril and fibronectin deposition, in agreement with a subsequent study utilizing mouse embryonic fibroblasts (Cain et al., 2016; Mead et al., 2022).

ADAMTS6 was shown to have a role in gap junctions in ventricular myocardium. *Adamts6*-deficient embryonic myocardium had a reduction of Cx43 staining, a predominant myocardial gap junction protein (Thomas et al., 1998; Gutstein et al., 2001; Prins et al., 2018). This was also evident in adult *Adamts6* ^+/−^ myocardium without a change in *Gja1* mRNA levels, which suggested post-transcriptional regulation (Prins et al., 2018). These findings point to the possible function of ADAMTS6 in prolonged QRS interval resulting from impaired myocardial connectivity.

**FIGURE 2:**
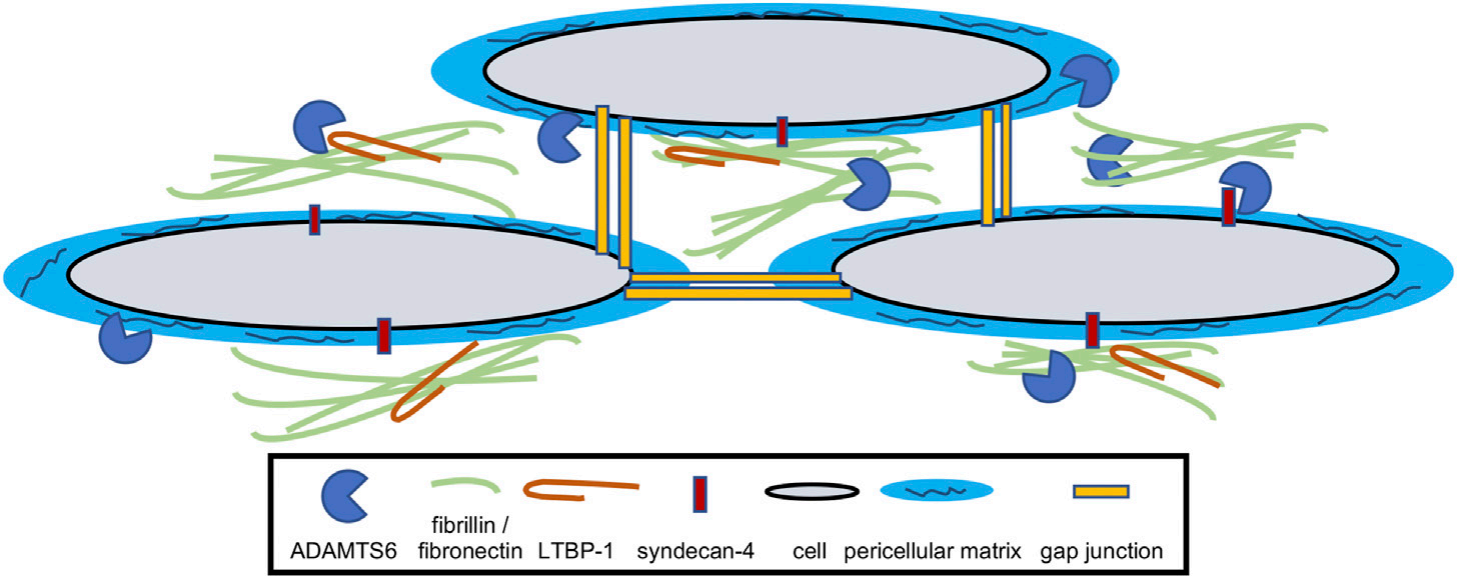
Model for the function of ADAMTS6. ADAMTS6 locates and functions within the extracellular matrix and has been shown to cleave fibrillin-1, fibrillin-2, fibronectin, LTBP-1 and syndecan-4. It directly or indirectly effects cellular gap junctions as well as pericellular matrix/glycocalyx quantity.

In summary, ADAMTS6 is a major component of the ECM and regulates several biological processes during development and disease (Figure 2). Further studies on patient diseases and biological samples will uncover additional regulatory network roles of ADAMTS6, which will generate supplementary scientific avenues and tools to reveal therapeutic potential. Understanding the full landscape of ADAMTS6 targets is necessary to unlock its potential in congenital malformations and disease.
